# Selecting Infants With Cryptorchidism and High Risk of Infertility for Optional Adjuvant Hormonal Therapy and Cryopreservation of Germ Cells: Experience From a Pilot Study

**DOI:** 10.3389/fendo.2018.00299

**Published:** 2018-06-05

**Authors:** Jorgen Thorup, Erik Clasen-Linde, Lihua Dong, Simone Hildorf, Stine Gry Kristensen, Claus Yding Andersen, Dina Cortes

**Affiliations:** ^1^The Department of Pediatric Surgery, Copenhagen University Hospital, Rigshospitalet, Copenhagen, Denmark; ^2^Faculty of Health and Medical Sciences, University of Copenhagen, Copenhagen, Denmark; ^3^The Department of Pathology, Copenhagen University Hospital, Rigshospitalet, Copenhagen, Denmark; ^4^Laboratory of Reproductive Biology, Section 5712, Juliane Marie Centre for Women, Children and Reproduction, Rigshospitalet, Copenhagen, Denmark; ^5^Section of Endocrinology, Department of Pediatrics, Copenhagen University Hospital Hvidovre, Hvidovre, Denmark

**Keywords:** cryptorchidism, germ cells, LHRH, cryopreservation of cells and tissues, orchiopexy, fertility

## Abstract

**Introduction:**

Orchiopexy for congenital cryptorchid testes is recommended between ½ and 1 year of age to preserve testicular germ cell maturation. Early operation is not enough to preserve fertility in 22 and 36% of cases. Aim of this study was to set up a protocol for optional adjuvant hormonal therapy after orchiopexy and thereafter cryopreservation of testicular biopsies from infants with bilateral cryptorchidism and high infertility risk.

**Materials and methods:**

We included 17 boys with bilateral cryptorchidism, normal FSH, and impaired germ cell number per tubular transverse section (G/T) in testicular biopsies at orchiopexy, 7 months to 3½ years old. Postoperatively, optional adjuvant LHRH (kryptocur^®^) 0.2 mg/0.1 mL 2× every second day in 16 weeks were offered. Ten boys were applicable for age matching according to parent’s choice of treatment regime and G/T. Five of them had kryptocur^®^, and five were controls. Repeat bilateral testicular biopsy evaluation and cryopreservation were offered to all boys 12 months after primary orchiopexy. For cryopreservation, tissue pieces were incubated with a cryoprotectant with a slow program freezing.

**Results:**

Two out of five kryptorcur^®^-treated boys normalized both the average G/T and the number of adult dark spermatogonia (Ad-S). Another kryptocur^®^-treated boy with initial low G/T and no Ad-S increased the G/T and achieved normal number of Ad-S at time of cryopreservation. In the control group, two patients reached only normal lower range regarding the G/T and the number of Ad-S. None of boys with less than average 0.2 G/T improved significantly, whether they were kryptocur^®^-treated or not.

**Conclusion:**

Based on literature and the present results, we recommend adjuvant LHRH treatment to boys with cryptorchidism and insufficient genuine gonadotropin stimulation at time of surgery, as these patients have high infertility risk. Cryopreservation should be an option in case of treatment failure of adjuvant LHRH. However, to avoid repeat surgery with biopsy, some parents may choose biopsy for cryopreservation at time of the initial bilateral orchiopexy, well informed that the procedure may only be truly indicated in 22 and 36% of the cases.

## Introduction

Today, orchiopexy for congenital cryptorchid testes is recommended within the first ½–1 year of life to preserve testicular germ cell maturation (1–4). In accordance with the strategy of early operation follow-up studies on adult men operated for cryptorchidism in childhood have shown significant improvement of fertility even in bilateral cases (5–7). However, these studies have not conclusively proven that early surgery will protect from azoospermia development.

For a subgroup of boys with cryptorchidism, early operation is not enough to preserve fertility. If, there is an underlying endocrinopathy causing inadequate maturation of the testis, merely putting the testis into the scrotum may not correct that endocrinopathy (4, 8). However, some of the reported endocrinopathies may be temporary and part of a maturational process. Specifically, there is incomplete evidence whether an appropriately time orchiopexy increases adult dark spermatogonia (Ad-S) numbers later similar to what LHRH may aid earlier on. One author showed that early and successful orchiopexy (before 9 months of age) could not prevent infertility development in 36% of cryptorchid males (1, 7, 9). Cortes et al. (10) found similarly that germ cell hypoplasia, which is germ cells per tubular cross section value below the lowest normal value for age, was present in 22% of the testes in 0- to 1-year-old boys with cryptorchidism. From about 15 months of age, germ cells may even start to lack and this process will progress (10, 11). Germ cell hypoplasia in both testes of prepubertal boys with cryptorchidism at time of orchiopexy generally leads to infertility in adulthood (12).

For more than a decade, Hadziselimovic and Hoecht have advocated that a group of boys with cryptorchidism and high risk of infertility could be identified in infancy according to histopathological evaluation of testicular biopsies showing low germ cell number and lack of Ad-S in the germinative epithelium (1, 2). Recently, Thorup et al. (13) proposed to combine the results of blood samples (gonadotropins and inhibin B) with histopathology (bilateral testicular biopsies during orchidopexy) to identify the group of patients at high risk of infertility. They reported that boys with normal gonadotropin levels and normal germ cell number have a good fertility prognosis and boys with increased gonadotropin levels may have testicular dysgenesis, and some of these boys may benefit from early surgery alone. However, boys with normal gonadotropin levels and a decreased total number of germ cells and decreased number of Ad-S have transient hypothalamus hypofunction and a poor fertility prognosis, as there was no gonadotropin response to the impaired histological state of seminiferous tubules as reflected either directly with the mean germ cell count per tubular transverse section or indirectly by the serum levels of inhibin B. The reason could be a significant prepubertal transient hypothalamus–pituitary–gonadal hypofunction (14). Impaired transformation of the neonatal gonocytes into type Ad-S during the first 12 months of age and subsequent apoptosis of germ cells may be a pathogenic factor. This transformation may be impaired if gonadotropins are insufficient and when the testis is undescended this lead to germ cell deterioration. Adjuvant hormonal treatment has been used to improve the fertility potential in such cases (15, 16).

For prepubertal infant boys with cryptorchidism, when full spermatogenesis is not yet ongoing, cryopreservation of immature testicular tissue might be an option to try to preserve their fertility while germ cells are still present in the cryptorchid testes, although the number of germ cells, as previously mentioned, is impaired (17, 18). Cryopreservation of testicular tissue has the advantage of preservation of integrated tissue. This allows to maintain cell-to-cell contact between spermatogonia and the neighboring cells, especially Sertoli cells, which are important for subsequent maturation of spermatogonia. We and another team have reported the freezing protocols of testicular tissue in prepubertal boys, both yielding good structural integrity (17, 19). Several centers have now established testicular tissue banking for prepubertal males at risk of infertility (20, 21). Young boys, before starting gonadotoxic cancer treatment (20, 21) or undergoing bilateral orchiopexy (21), are offered the option of testicular biopsy and cryopreservation under the same general anesthesia for either port-a-cath-insertion (for chemotherapy) or orchiopexy surgery. A biopsy of about 0.35 mm^3^, either 5% of testicular volume, is considered as a sufficient amount of tissue for culture and transplantation. Although testicular tissue preservation offers the prospect of realistic applications, fertility restoration after cryopreservation has not yet been successful in humans. Nevertheless, promising results of restoration of fertility from donor stem cells have been achieved in animals (22).

The aim of this study was to set up and evaluate a useful protocol for optional adjuvant hormonal therapy and cryopreservation of testicular biopsies from infant boys with cryptorchidism and high risk of infertility. We hypothesize that this strategy is relevant, because within a 20-year period the technique of restoration of fertility from autologous donor stem cells has been achieved in humans.

## Materials and Methods

### Testicular Biopsies

Testicular biopsies at time of orchiopexy were taken and evaluated according to a previous described method (10). All tissue specimens were fixed in Stieve’s solution, embedded in paraffin, and 2-µm sections were stained with hematoxylin-eosin, CD99 (MIC-2) and PLAP. In blinded fashion, the total number of germ cells (S/T), including gonocytes and Ad-S, per tubular transverse section was measured from at least 100 tubular transverse sections. For every patient, the mean adult dark spermatogonia per tubule (Ad/T) and mean-S/T were found. The mean-S/T was considered normal when the value was at least 1.0 at birth, 0.65 at 6 months and 0.38 in 1- to 4-year-old boys, based on our previously published normal material (10). The total number of Ad/T for each patient was stratified into present (greater than 0.02) or abnormal (0.02 or less).

### Hormonal Assays

Blood samples were obtained by venipuncture between 8:00 and 11:00 a.m. Serum samples were separated from the clot by 10 min of centrifugation at 2,000 × *g*. Serum was stored at −80°C until analysis. Serum inhibin B levels were measured using a commercial available inhibin B ELISA kit (Serotec Ltd., Oxford, UK) with research kit as recommended by the manufactory instructions. The lower detection limit was 5 pg/mL, and the measurements were made in duplicate.

LH and FSH were measured by sandwich electrochemiluminescence immunoassay. The lowest value of FSH to be measured was 0.05 IU/L. Normal median: 0.5 IU/L and range: 0.1–1.4 IU/L. The lowest value of LH to be measured was also 0.05 IU/L. Normal median: 0.1 IU/L and range: 0.05–0.3 IU/L.

FSH more than 1.4 IU/L was considered as a high serum value for this age group.

### Patients and Setup

We offered boys age 7 months to 3½ years old with bilateral cryptorchidism to join the study if they met the inclusion criteria.

In order for patients to be qualified for inclusion, the average germ cell count per tubular transverse section should be between 0.05 and 0.40. PLAP positive germ cells should be present in the biopsy. The serum FSH level should not be increased.

After 3 months postoperatively, when testes should be in scrotum and the hormone profile and testicular histology was evaluated, the patients were offered adjuvant hormonal treatment with LHRH (kryptocur^®^) 0.2 mg/0.1 mL 2× every second day in 16 weeks (dosing according to consensus conference, Cortes and Hadziselimovic; Liestal 2009). Patients, with matched germ cell number in testicular biopsies, who did not choose hormonal treatment were used for controls. Repeat bilateral testicular biopsy for histology and cryopreservation were offered to all the patients 12 months after the primary orchiopexy.

Cryopreservation was performed according to the following protocol: tissue pieces were incubated with a cryoprotectant (5% DMSO) with a slow program freezing.

### Statistics

Non-parametric statistics were used for analyses: Mann–Whitney test was used to asses statistical significance, and two-sited *p* values less than 0.05 were considered significant.

#### Ethics

The study was conducted according to the Helsinki II declaration, and informed consent was obtained from the parents of the patients. The study received approval from the ethics committee of Copenhagen (H-2-2012-060.anm.37655) and Danish Medicines Agency (SST jr. nr. LMST-2012083184).

## Results

Seventeen boys with bilateral cryptorchidism had testicular biopsies taken for cryopreservation. Ten boys were applicable for age matching according to parent’s choice of treatment regime and histopathological evaluation of germ cell status. These boys were included in the study. Five of them had kryptocur^®^ treatment and five were controls. None had any associated anomalies.

**Figure 1:**
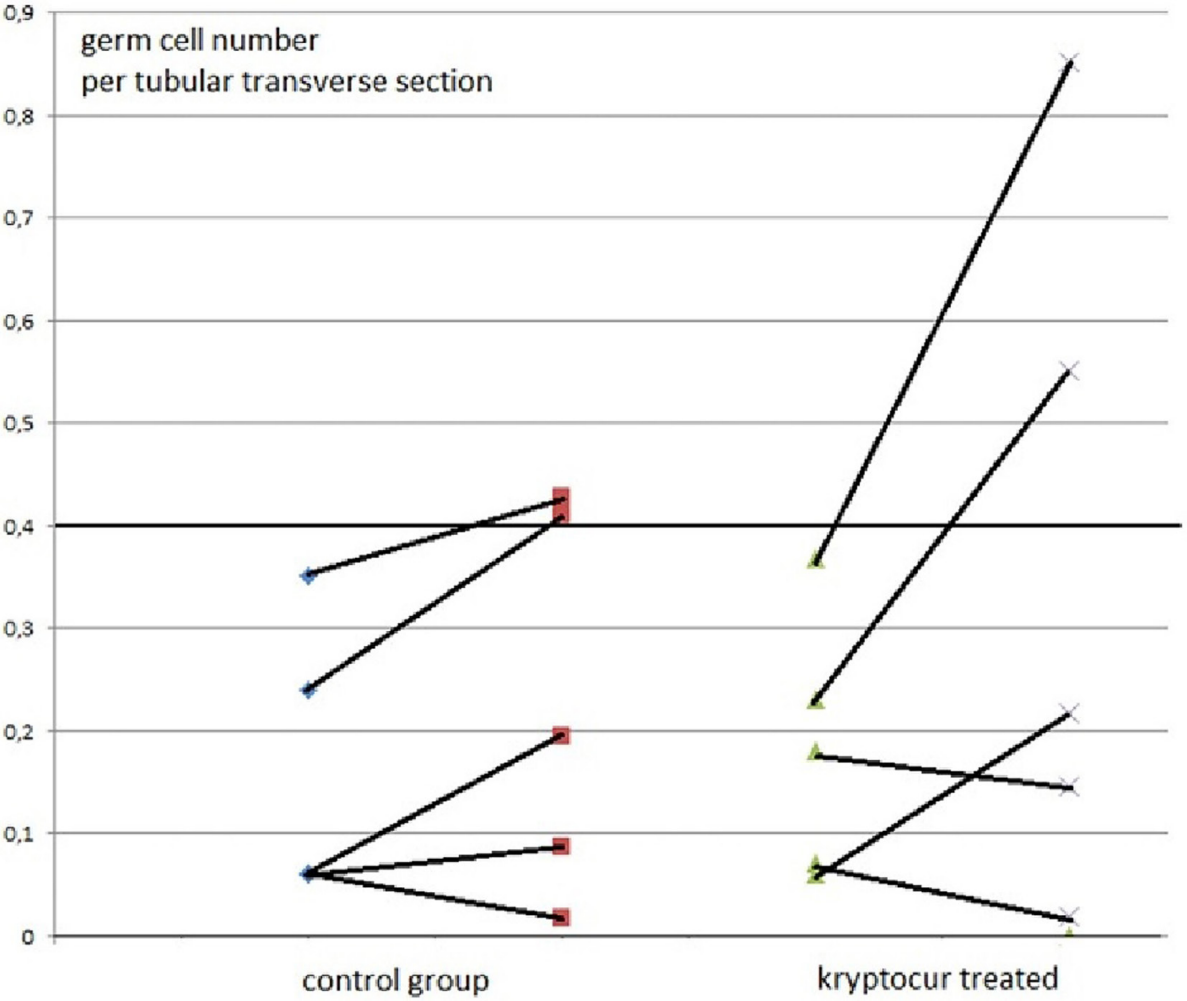
The average number of germ cells per tubular transverse section from 10 boys with bilateral cryptorchidism at time of orchiopexy and 1 year after, at time of cryopreservation. The individual results of primary and follow-up biopsies from five kryptorcur^®^ treated and five bilateral controls without hormonal treatment aged 10 months to 3 years are connected with lines [there was no age difference between groups (*p* = 0.92)].

At inclusion, there were no differences between the two groups in respect of age (*p* = 0.92), inhibin-B level (*p* = 0.69), and the average germ cell count per tubular transverse section (*p* = 0.40) (Figure 1).

Two out of five kryptorcur^®^-treated boys normalized completely as well the average germ cell count per tubular transverse section as the number of Ad-S. One other kryptocur^®^-treated boy with an initial germ cell count per tubular transverse section of 0.06 and no Ad-S increased the germ cell count and achieved normal number of Ad-S at time of cryopreservation. In the control group, two patients reached normal lower range regarding the average germ cell count per tubular transverse section and the number of Ad-S (Figure 1). None of the boys with less than average 0.2 germ cell number per tubular transverse section improved significantly, whether they were kryptocur^®^ treated or not (Figure 1). There were no surgical complications after the re-biopsy procedure. All specimens for cryopreservation were prepared, frozen, and stored according to the protocol without complications. The median and (range) hormonal serum levels at time of orchiopexy and re-biopsy for cryopreservation were FSH: 0.7 (0.6–1.4) and 0.4 (0.1–1.7) IU/L, respectively; LH: 0.1 (0.05–0.4) and 0.1 (0.05–0.4) IU/L, respectively; inhibin B: 114 (17–300) and 55 (28–132 pg/mL), respectively.

## Discussion

This is the first study on cryopreservation of testicular tissue from infant boys with documented high risk of infertility after treatment for cryptorchidism. Although the study sample is very modest, we demonstrate important results, which are useful for further investigations. Since 2/5 (and one partial) of LHRH-treated patients responded, adjuvant LHRH treatment to cryptorchid boys with insufficient genuine gonadotropin stimulation or in patients with low germ cell count or number of Ad-S by testicular biopsy should be instituted at time of surgery. However, since 2/5 control patients had almost similar improvements in germ cell counts, one could also argue that the study does not show any significant difference between treated and control patients. Thus, the conclusion should be that a larger sample size is needed to fully discern whether the germ cell count improvement is truly only seen in LHRH-treated patients. Our findings are evidently in agreement with the research by Hadziselimovic, who states that if germ cell count per tubular transverse section is below 0.2, a majority of patients will develop infertility irrespective of whether they had only surgery or hormonal treatment before orchiopexy (9). So, the only realistic way to achieve biological paternity for these patients may be through cryopreservation of testicular tissue. Great challenges in this respect are obvious. We have previously clearly shown that infant testicular biopsies tolerate the freezing procedure (19). In that study the morphology of the fresh and frozen–thawed samples was similar, with well-preserved seminiferous tubules and interstitial cells. A similar picture appeared after 2 weeks of culture, but a few of the cultured biopsies contained small necrotic areas. The presence of spermatogonia was verified by c-kit-positive immunostaining. Production of testosterone and inhibin B (ng/mm^3^ testis tissue) in the frozen–thawed pieces were on average similar to that of the fresh samples (19). However, although PLAP positive germ cells were present in our present selected material, possibly representing germ cells with stem cell properties and with preserved ability for germ cell transformation (23, 24), presence of Ad-S was not identified in all biopsies for cryopreservation. Cultivation of a spermatogonia cell population is a prerequisite for later restoration of fertility from an autologous donor. So, development in our laboratory of a technique for testicular autograft enzymatically dispersed leading to single spermatogonia stem cell suspension is needed and is now in process.

**Figure 2:**
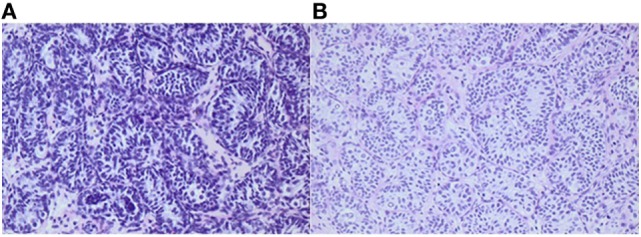
Histology of testicular biopsies from a boy when he was 1¼ and 2¼ years old, respectively. After bilateral orchiopexy for cryptorchidism, 4 months of adjuvant LHRH was given. Average germ cell number per tubular transverse section increased from 0.27 **(A)** to 0.55 **(B)**. Adult dark spermatogonia (Ad-S) are noted in the re-biopsy **(B)**. The Ad-S are characterized as large gem cells with pale cytoplasm. In the nuclei, a vacuole is observed which appears in the sections as a circular light area. Informed consent to show the pictures was given by the parents.

Although there are too few patients in our study to show any statistical significance our data may support the studies in a recent review implicating that LHRH treatment increases the germ cell number in cryptorchid testes (16): “Hadziselimovic and Herzog reported in 1997 that the luteinizing hormone-releasing hormone analog, Buserelin^®^, administered intranasally every other day for 6 months following successful orchidopexy, appeared to have a long-lasting, positive effect on germ cells (15). In another study, Huff et al. (25) reported that GnRH treatment after orchidopexy improved total germ cell counts in 75% of the patients. These results have partly been confirmed by other groups. Schwentner et al. (26) gave intranasal GnRH 1.2 mg/day for 4 weeks preoperatively in 21 cases versus orchidopexy only in 21 controls. The mean number of spermatogonia per tubule was 1.11 in unilateral and 0.96 in bilateral cases versus 0.47 and 0.56, respectively, in controls. Jallouli et al. (27) prospectively assigned a total of 24 boys, 12–123 months old (median 34.5), with 24 UDT into two groups during a 24-month period. The patients were randomized to receive either orchidopexy alone (*n* = 12) or orchidopexy combined with neoadjuvant GnRH therapy (kryptocur^®^) (*n* = 12) as a nasal spray for 4 weeks at 1.2 mg/day. In both groups, testicular biopsies were performed at orchidopexy, and the number of germ cells per tubule was determined. The mean number of germ cells per tubule in the group treated with GnRH before surgery was significantly greater (0.88 ± 0.31) than in the group without hormonal stimulation (0.49 ± 0.52; *p* = 0.02). Zivkovic et al. (28) also found that hormonal therapy (Buserelin^®^) improved the histopathology of the abnormal contralateral descended testis in unilateral cryptorchidism without harming the germ cells.” Vincel et al. (29) presented recently a randomized study of 10 boys 8 months to 5 years old. Boys with high infertility risk (no Ad-S in the testicular biopsy) were randomly divided into two groups. First group underwent just the second orchidopexy without any hormonal treatment. The second group received intranasal LHRH (Buserelin^®^) therapy for the period of 6 months followed by second orchidopexy. Biopsy was taken from both groups during the second surgery. Five boys in each arm were included. There was no difference in the mean number of germ cells per tubule at the first surgery between the first and the second groups. Only the second group showed statistically significant increase in the number of germ cells per tubule: medians from 0.11 to 0.42 (*p* = 0.04). There were no Ad spermatogonia in both groups in the first biopsy. They were detected only in patients from the second group, who had adjuvant hormonal therapy, in the second biopsy (*p* = 0.008). These findings are very similar to our present results (Figures 1 and 2).

So, adjuvant hormonal therapy stimulating gonadotrophins may probably have a positive effect on germ cell maturation (16). Due to our previous findings that LHRH in recommended doze may harm the germ cells in young infancy (30), we have chosen a lower doze and a longer treatment period in the present study. Further pharmaceutical safety studies and randomized placebo controlled efficiency studies are needed to find the optimal adjuvant treatment regime. However, hormonal treatment should be restricted to boys with genuine insufficient gonadotropin stimulation, which may also include unilateral cryptorchidism with bilateral disease. The hormonal changes from orchiopexy to time of re-biopsy are in accordance with the expected changes related physiological decline of serum values seen after the minipuberty (31). The weakness of the study is related to the modest sample size and the lack of a randomized design. Furthermore, there is no guaranty that the development of technique for restoration of fertility from autologous donor stem cells will be successful within 15–20 years, although very promising rodent studies have recently been published (32).

In 1994, a first report showed that spermatogonia stem cell transplantation restored spermatogenesis, resulted in functional sperm and subsequently gave rise to normal offspring in mice (33, 34). Till now, spermatogonia stem cell transplantation has been successful in various species such as pig, bovine, and monkey (35–37). In addition, it has been shown that spermatogonia stem cells from humans and other species could settle in their niche on the basal membrane of testis tubules after transplantation into immunodeficient mice but currently there is no experience from transplanting spermatogonia stem cells to humans (38–41).

## Conclusion

Based on the literature and the present results we recommend adjuvant LHRH treatment to boys with cryptorchidism and insufficient genuine gonadotropin stimulation at time of surgery, as these patients have high risk of infertility. But a larger study sample size is needed to fully discern whether the germ cell count improvement is truly only seen in LHRH-treated patients. Cryopreservation should be an option in case of treatment failure of adjuvant LHRH. However, to avoid repeat surgery with biopsy, it may be more attractive for some parents to choose biopsy for cryopreservation at time of the initial bilateral orchiopexy, well informed that the procedure may only be truly indicated in 22 and 36% of the cases.

## Ethics Statement

The study was conducted according to the Helsinki II declaration, and informed consent was obtained from the parents of the patients. The study received approval from the ethics committee of Copenhagen (H-2-2012-060.anm.37655) and Danish Medicines Agency (SST jr. nr. LMST-2012083184).

## Author Contributions

JT designed study, recruited and treated patients, wrote first manuscript draft, and discussed and revised manuscript. EC-L evaluated histology and discussed and revised manuscript. LD and SK did cryopreservation an discussed and revised manuscript. SH and CA discussed and revised manuscript. DC counted germ cells and discussed and revised manuscript.

## Conflict of Interest Statement

The authors declare that the research was conducted in the absence of any commercial or financial relationships that could be construed as a potential conflict of interest.
